# Group cognitive stimulation therapy for people with intellectual disability and dementia: feasibility randomised controlled trial

**DOI:** 10.1192/bjo.2025.10764

**Published:** 2025-08-01

**Authors:** Afia Ali, Cheryl Francis, Sarah Hoare, Joanna Carter, Nia Goulden, Caroline S. Clarke, Georgina Charlesworth, Zoe Hoare, Danny Acton, Shafia Khanum, Akinwande Onafalujo, Adebayo Jejeloye, Kate Brackley, Elisa Aguirre, Aimee Spector

**Affiliations:** Wolfson Institute of Population Health, Queen Mary University of London, London, UK; Department of Clinical, Educational & Health Psychology, University College London, London, UK; North Wales Organisation for Randomised Trials in Health and Social Care, Bangor University, Bangor, UK; Research Department of Primary Care and Population Health, University College London, London, UK; Research and Development, North East London NHS Foundation Trust, London, UK; Millenium Centre, Cheshire and Wirral Partnership NHS Foundation Trust, Chester, UK; Services for People with Learning Disability, East London NHS Foundation Trust, London, UK; British Institute of Learning Disabilities, Birmingham Research Park, Edgbaston, UK; Psychology Department, Universidad Europea de Madrid, Madrid, Spain

**Keywords:** Cognitive stimulation therapy, intellectual disability, dementia, randomised controlled trial, feasibility

## Abstract

**Background:**

Group cognitive stimulation therapy (CST) has been shown to improve cognition and quality of life of people with dementia in multiple trials, but there has been scant research involving people with intellectual disability and dementia. This study aimed to assess the feasibility of conducting a randomised controlled trial of group CST for this population.

**Aims:**

To assess the feasibility of participant recruitment and retention, the appropriateness of outcome measures, and the feasibility of group CST (adherence, fidelity, acceptability), as well as the feasibility of collecting data for an economic evaluation.

**Method:**

Participants were recruited from six National Health Service trusts in England and randomised to group CST plus treatment as usual (TAU) or TAU only. Cognition, quality of life, depression, and use of health and social care services were measured at baseline and at 8–9 weeks. Qualitative interviews with participants, carers and facilitators were used to explore facilitators of and barriers to delivery of CST. Trial registration number: ISRCTN88614460.

**Results:**

We obtained consent from 46 participants, and 34 (73.9%) were randomised: 18 to CST and 16 to TAU. All randomised participants completed follow-up. Completion rates of outcome measures (including health economic measures) were adequate; 75.7% of sessions were delivered, and 56% of participants attended ten or more. Fidelity of delivery was of moderate quality. CST was acceptable to all stakeholders; barriers included travel distance, carer availability and sessions needing further adaptations. The estimated cost per participant of delivering CST was £602.

**Conclusions:**

There were multiple challenges including recruitment issues, a large dropout rate before randomisation and practical issues affecting attendance. These issues would need to be addressed before conducting a larger trial.

People with intellectual disability are at increased risk of developing dementia, often with earlier onset, compared with the general population.^1,2^ The consequences of dementia in people with intellectual disability include faster progression and elevated mortality rates.^1^ The National Institute for Health and Care Excellence (NICE) guidelines for dementia (NG97) emphasise person-centred care and psychosocial interventions.^3^ People with intellectual disability are often excluded from person-centred care initiatives, and this extends to the management of dementia.^4–6^ Two published reviews of psychosocial interventions for individuals with intellectual disability and dementia found a limited number of direct therapeutic interventions with the individual, with interventions being largely carried out with carers.^2,7^

Cognitive stimulation therapy (CST) is a NICE-recommended manualised psychosocial intervention for individuals with mild to moderate dementia.^3^ It is a 14-session group intervention incorporating 18 key principles including mental stimulation and person-centredness, with each session covering a different theme (e.g. physical games, childhood and food). Clinical trials worldwide have shown consistent improvements in a range of outcomes including cognition, language, working memory, depression, communication, neuropsychiatric symptoms and quality of life.^8^ However, the presence of intellectual disability has been an exclusion criterion for the majority of published CST trials.^9^

Our team previously evaluated individualised CST in 40 individuals with dementia and intellectual disability and concluded that it was feasible and acceptable, with improved quality of life at 21 weeks but no changes in adaptive functioning or cognition.^10^ However, significant barriers were identified, including some activities being unsuitable for individual needs or cognitive ability and carers struggling to deliver individualised CST within their busy work schedules. There have been no randomised controlled trials (RCTs) of group CST in people with intellectual disability and dementia. As group CST is recommended by NICE for people with dementia,^3^ exploration of group CST for those with intellectual disability is warranted.

In this study, we aimed to assess the feasibility and acceptability of group CST for people with intellectual disability and dementia compared with treatment as usual (TAU), with a view to informing the design of a future definitive RCT. The objectives were to assess: (a) feasibility of recruitment and retention of participants; (b) suitability of outcome measures; (c) feasibility of the CST intervention (acceptability, adherence and fidelity, and serious adverse events); and (d) feasibility of collecting data to inform a future health economic analysis. The study protocol has been published and provides further information about our methods and analysis.^11^

## Method

### Design

This was a single-blind feasibility RCT with a qualitative evaluation. The study was randomised to enable us to assess whether participants were willing to take part in a randomised study and to test randomisation procedures before a full RCT. No formal power calculation was conducted. We set a target sample size of 50 to achieve adequate precision around our expected retention rate of 75% (95% CI: 62 to 86%). The authors assert that all procedures contributing to this work comply with the ethical standards of the relevant national and institutional committees on human experimentation and with the Helsinki Declaration of 1975, as revised in 2013. All procedures involving human subjects/patients were approved by the East of England – Essex Research Ethics Committee (reference number: 21/EE/02/47).

### Recruitment and procedures

Participants were recruited from community intellectual (‘learning’) disability teams from six National Health Service (NHS) trusts in England. To recruit to the study, professionals screened case-loads for possible participants and approached them via their carer. If interested in participating, potential participants were provided with an information sheet, and a researcher subsequently contacted the individual and their carer to discuss the study and to assess capacity to consent to taking part. If the individual agreed to participate, written consent was obtained. If they lacked capacity, a relative or friend (personal consultee) was approached, provided with a consultee information sheet and asked to sign a declaration form. If the individual did not have a personal consultee, a nominated consultee (e.g. a clinician not involved in the study) was approached instead. Carers completing proxy and/or informant measures were asked to provide consent to take part in the study. A demographic questionnaire was completed at baseline and included information about the participant’s age, gender, ethnicity, level of intellectual disability, severity and type of dementia, living arrangements, comorbid medical conditions and medication. Information about the carer’s age, gender, ethnicity and relationship or role was also collected. If a different carer was present at the follow-up appointment, their details were recorded at that appointment.

The inclusion criteria were: (a) premorbid mild or moderate intellectual disability (based on service records); (b) age 18 years or over; (c) clinical diagnosis of mild or moderate dementia (based on clinical notes); (d) ability to provide informed consent or (if the participant lacked capacity) availability of a personal consultee who has agreed for the individual to participate; (e) ability to communicate in English. The exclusion criteria were: significant visual or hearing impairment, physical illness or disability, or significant behavioural problems (e.g. aggressive behaviour) that could affect participation.

### Randomisation and blinding

Participants were randomised at each site with a minimum of five participants. Randomisation was undertaken by the coordinating trials unit (North Wales Organisation for Randomised Trials in Health and Social Care) using a dynamic adaptive algorithm via a secure online interface. Randomisation was stratified by site or centre using a 1:1 allocation ratio. Participants and carers were informed of their group allocation by an unblinded researcher. Research assistants conducting quantitative follow-up assessments were blind to group allocation.

### CST intervention arm

Participants in the intervention arm were expected to receive 14 face-to-face sessions delivered over 7 weeks. The target group size was between three and five participants, that is, the groups were smaller than those used for CST in people without intellectual disability. This was because people with intellectual disability and dementia are more likely to need support and reasonable adjustments to enable them to participate owing to communication and sensory impairments. Groups were facilitated by health professionals from the participating community intellectual disability team. These professionals had to have experience of working with people with intellectual disability and included psychologists, psychiatrists, occupational therapists and nurses. The groups took place within clinical services (and not at participants’ usual day care setting). Facilitators received standard CST training (1 day) followed by an additional session on adapting CST for this population. They used the CST treatment manual with a supplement outlining adaptations for individuals with intellectual disability, which was developed for the study. Examples of adaptations included simplification of activities, replacement of words with pictures where possible and activities that provided multisensory stimulation. The intervention group continued to have access to TAU (see below).

### TAU arm

Participants allocated to the TAU arm received their usual care, including support from psychiatrists, nurses and psychologists; access to day care; and medication including antidementia (cognitive-enhancing) medication (e.g. acetylcholinesterase inhibitors).

### Outcome measures

Outcome assessments were conducted at baseline (within 2 weeks before randomisation) and follow-up (8–9 weeks post-randomisation) via face-to-face assessments. The following measures were completed:

- the Severe Impairment Battery (SIB) to assess cognitive functioning;^12^
- the Dementia Questionnaire for people with Learning Disabilities (DLD), an informant measure used to assess cognitive and social functioning;^13^
- the Quality of life in Alzheimer’s Disease (QOL-AD) – proxy version, an informant measure of the participant’s quality of life;^14^
- the Glasgow Depression Scale for People with Learning Disability (GDS-LD) proxy version, an informant measure used to assess the presence of depressive symptoms;^15^
- EQ-5D quality of life questionnaires: proxy version,^16^ completed by carers on behalf of participants; and EQ-5D-5L self-report, completed by participants in the initial phases of the study and replaced partway with a modified version of EQ-5D-3L for adults with intellectual disability;^17,18^
- a modified version of the Client Service Receipt Inventory (CSRI), tailored to this patient group and context in collaboration with patients, parents and carers, to collect information about health and social care resources.^19^

### Feasibility outcomes

The feasibility outcomes were assessed according to ‘Go/Review/Stop criteria’, which were used to determine the success of the trial and whether a future definitive RCT was feasible. The specific criteria are listed in Table 1.

**Table 1:** Stop/Go criteria for the feasibility outcomes

Feasibility outcome	Description	Target
Recruitment of participants	Adequate recruitment	Go: ≥38 participants were recruited to the trial; Review: 25–37 participants were recruited; Stop: <25 participants were recruited.
	Eligibility rate	Go: ≥75% of referred participants were eligible; Review: 50–74% of referred participants were eligible; Stop: <50% of participants were eligible.
	Consent/recruitment rate	Go: ≥75% of eligible participants were recruited; Review: 50–74% were recruited; Stop: <50% of eligible participants were recruited.
Retention of participants in the study		Go: ≥75% of recruited participants completed the trial; Review: 50–74% of those recruited completed the trial; Stop: <50% completed the trial.
Suitability of outcome measures	Completion rates of outcome measures	Go: ≥85% of participants completed each measure at an acceptable level at each time point; Review: this was achieved by 70–84% of participants; Stop: it was achieved by <70%.
	Sensitivity of outcome measures to change	Go: outcome measures show change in favour of the intervention; Review: only one or two outcome measures show change in favour of intervention; Stop: no outcome measures show change in favour of intervention arm.
Adherence	Percentage attending at least ten sessions	Go: 75% of participants attended at least ten delivered intervention sessions; Review: 50–74% attended at least ten delivered sessions; Stop: fewer than 50% of participants attended ten sessions.
	Percentage of sessions delivered by facilitators	Go: 75% of all intervention sessions were delivered by facilitators; Review: 50–74% of sessions were delivered; Stop: <50% of sessions were delivered.
Adverse events	Number and type of adverse events	Go: no serious adverse events relating to CST; Review: minor adverse events relating to CST; Stop: at least one serious adverse event relating to CST.
Fidelity	Extent to which intervention components were delivered (percentage score)	Go: 80–100% adherence to the fidelity checklist (high fidelity); Review: 51–79% adherence to the fidelity checklist (moderate fidelity); Stop: 50% or lower adherence to fidelity checklist (low fidelity).
Acceptability	Satisfaction with CST	Based on qualitative interviews and includes perceived benefits and negative effects.

CST, cognitive stimulation therapy.

Recruitment and retention were assessed using the following criteria:

- adequate recruitment, defined as the number of participants recruited to the trial;
- eligibility rate, defined as the percentage of participants who met the eligibility criteria from those referred;
- consent/recruitment rate, defined as the percentage of participants recruited to the trial of those eligible;
- retention, defined as the number of participants completing the trial (follow-up assessment) from those recruited.

The suitability of study outcome measures was assessed on the basis of:

- completion rates of outcome measures, defined as the proportion of participants completing outcome measures at baseline and follow-up; and
- whether outcome measures were sensitive to change in the population.

The feasibility of the CST intervention was assessed as follows.

- Adherence of CST: overall attendance among CST group participants was determined on the basis of group attendance registers completed by facilitators and percentages of sessions delivered by facilitators according to session logs.
- Fidelity of intervention delivery: fidelity was measured using a previously developed fidelity checklist, adapted for the study and based on CST principles and core intervention components.^20^ A four-point Likert scale was used to rate the extent to which each CST principle had been successfully incorporated into the session. The five intervention components (orientation, current affairs activity, main activity, use of themed therapy resources and obtaining feedback) were rated on a binary scale (‘1’ if a component had been completed and ‘0’ if it had not). The fidelity checklist was completed by group facilitators after each session. An independent observer listened to 50% of the available audio recordings of sessions and rated the sessions using the same checklist. A total fidelity score and a percentage fidelity score (obtained fidelity score/maximum checklist score (minus items that were non-applicable) × 100%) was calculated for each session for both facilitator and observer ratings. A percentage score of 80–100% adherence to the fidelity checklist was considered to indicate high fidelity, whereas 51–79% indicated moderate fidelity, and 50% or less indicated low fidelity.^21^
- Acceptability of CST: semi-structured interviews were conducted with group participants, carers and facilitators to assess acceptability and satisfaction with the intervention and to understand some of the barriers and enablers affecting feasibility outcomes such as retention and adherence. All group participants and carers and group facilitators were eligible for participation in qualitative interviews. Group participants were interviewed if available on the day of one of the final two sessions (to maximise likelihood of recall of the group experience). A Talking Mats approach was employed for participants with intellectual disability, as this is appropriate for use with individuals with a basic level of communication.^22,23^ Symbols and/or images were used for each question and placed on a visual scale to represent the following responses: ‘Like’, ‘Unsure’ or ‘Don’t Like’. Participants were asked about their views relating to the activities, frequency and duration of sessions, and being with others; carers were asked about positive and negative consequences of attending groups for the participant; and facilitators were asked about their experiences of preparing and delivering the sessions. Carers and facilitators were also asked to provide suggestions for improvements. Interviews were audio-recorded. Carer and facilitator interviews (up to 1 h) took place remotely and were recorded via Microsoft Teams. All the interviews were transcribed verbatim.
- Adverse events: numbers of serious adverse events during the study and whether they were related to the intervention were recorded.

Health-related quality of life was assessed using the EQ-5D-5L proxy^16^ at baseline and at follow-up, completed by the carer on behalf of the participant. The same questionnaire was administered to participants for them to self-complete during the early part of the study. Feedback suggested that this measure was hard for participants to complete; therefore, it was changed to the modified EQ-5D-3L for adults with learning/intellectual disabilities, which became available for piloting during the study.^17,18^ This measure has been specially adapted for use with individuals with intellectual disability (simplified text and easy-to-read images) with permission of the EuroQoL group. All participants were asked to complete the same measure at both time points; that is, they were asked either to complete the EQ-5D-5L at both time points, or to complete the modified EQ-5D-3L at both time points. To examine the feasibility of collecting healthcare and social care resource use, a modified CSRI^19^ questionnaire was used. This captured use of services related to the intervention, TAU in both arms, and other treatment pathway costs, including primary, community and hospital care, medications, and use of social services (funded by government or privately). The cost of the group CST intervention was estimated from information provided by staff at sites, which included staff time for training, facilitators running the session and resources purchased for the groups. As this was a feasibility study, we did not calculate overall costs or quality-adjusted life-years, and no formal comparisons were made between the groups; the purpose was to inform the design of a future definitive RCT and not to perform a cost-effectiveness analysis.

### Statistical analysis

Statistical analysis was conducted using Stata version 16 for Windows. Participant flow data were analysed, and values for eligibility, recruitment, attrition and withdrawal rates are presented, including reasons for ineligibility and withdrawals. Descriptive statistics were used to describe the data (e.g. mean and standard deviation for continuous data and counts and percentages for categorical data).

Outcome data were analysed using analysis of covariance. Adjusted means (adjusted for baseline values) were obtained, and 95% confidence intervals were calculated. Intraclass correlation coefficients were calculated to take into account the effects of clustering, which would need to be adjusted for in a sample size calculation.

Interrater agreement between the facilitator- and observer-reported fidelity scores was calculated using percentage agreement and the weighted kappa statistic (*κ*). We used thresholds of *κ* > 0.4 for moderate and κ > 0.6 for good interrater reliability.^24^

### Qualitative analysis

A thematic analysis was used to analyse data from facilitator and carer interviews. This analysis method combined inductive and deductive coding approaches^25,26^ to identify relevant themes related to acceptability, as well as barriers to and facilitators of the intervention. Participant interviews were analysed using basic content analysis.^27^

### Changes from the published protocol

There were a few changes to the analysis following publication of the study protocol.^11^ In our results, we now include the percentage of sessions delivered by facilitators. The assessment of fidelity was amended to include coverage of CST principles as well as intervention components; therefore, the criteria for assessment were updated to reflect this change. Owing to time constraints, we were unable to interview participants and carers in the control arm.

## Results

### Participant characteristics

In the whole sample, the mean age of participants was 61.9 years (s.d. 8.5), 61.8% were male, and most were of White British ethnicity (88.2%). Nineteen (55.9%) had a moderate learning disability, 55.9% had Alzheimer’s dementia, 50% had mild dementia and half were taking antidementia medication. Most lived in 24-h supported housing (76.5%). The carers who completed baseline measures were mostly paid carers (85.2%) with a mean age of 45.3 years (s.d. 12.9), and were mostly female (70.4%) and of White English British ethnicity (55.6%). Details of the sample’s demographics according to group allocation are presented in Table 2. The CST arm also had more male individuals (66.7 *v*. 56.3%) and more people of Black and minority ethnic origin (16.7 *v*. 6.2%).

**Table 2:** Participant demographics at baseline in the intervention (CST) and TAU arms

Characteristic	TAU *N* = 16, *n* (%)	INT *N* = 18, *n* (%)	Total *N* = 34, *n* (%)
Gender			
Male	9 (56.3)	12 (66.7)	21 (61.8)
Female	7 (43.8)	6 (33.3)	13 (38.2)
Prefer not to say and/or other	0 (0)	0 (0)	0 (0)
Severity of intellectual disability			
Mild	7 (43.8)	8 (44.4)	15 (44.1)
Moderate	9 (56.3)	10 (55.6)	19 (55.9)
Ethnicity			
White (any White background)	16 (100)	16 (88.8)	32 (94.1)
Other	0 (0)	2 (11.1)	2 (5.9)
Accommodation			
Lives with relative	1 (6.3)	2 (11.1)	3 (8.8)
Lives in supported housing (<24 h)	1 (6.3)	1 (5.6)	2 (5.9)
Lives in supported housing (24 h)	12 (75.0)	14 (77.8)	26 (76.5)
Lives in residential care	0 (0)	1 (5.6)	1 (2.9)
Lives in nursing home	1 (6.3)	0 (0)	1 (2.9)
Type of dementia			
Alzheimer’s	11(68.8)	8 (44.4)	19 (55.9)
Vascular	3 (18.8)	2 (11.1)	5 (14.7)
Mixed Alzheimer’s and vascular dementia	0 (0)	2 (11.1)	2 (5.9)
Not known	2 (12.5)	5 (27.8)	7 (20.6)
Stage of dementia			
Mild	9 (56.3)	8 (44.4)	17 (50.0)
Moderate	7 (43.8)	7 (38.9)	14 (41.2)
Not known	0 (0)	3 (16.7)	3 (8.8)
Hearing problems			
One or both ears	5 (31.3)	7 (38.39)	12 (35.3)
Visual problems			
One or both eyes	7 (43.8)	10 (55.6)	17 (50.0)
Mobility problems			
Uses wheelchair	4 (25.0)	3 (16.7)	7 (20.6)
Uses walking frame and/or other mobility aid	2 (12.5)	3 (16.7)	5 (14.7)
Epilepsy or seizures			
Yes	2 (12.5)	3 (16.7)	5 (14.7)
Antidementia medication			
Yes	8 (50.0)	9 (50.0)	17 (50.0)

CST, cognitive stimulation therapy; TAU, treatment as usual; INT, intervention.

### Feasibility outcomes

#### Recruitment and retention of participants

**Fig. 1:**
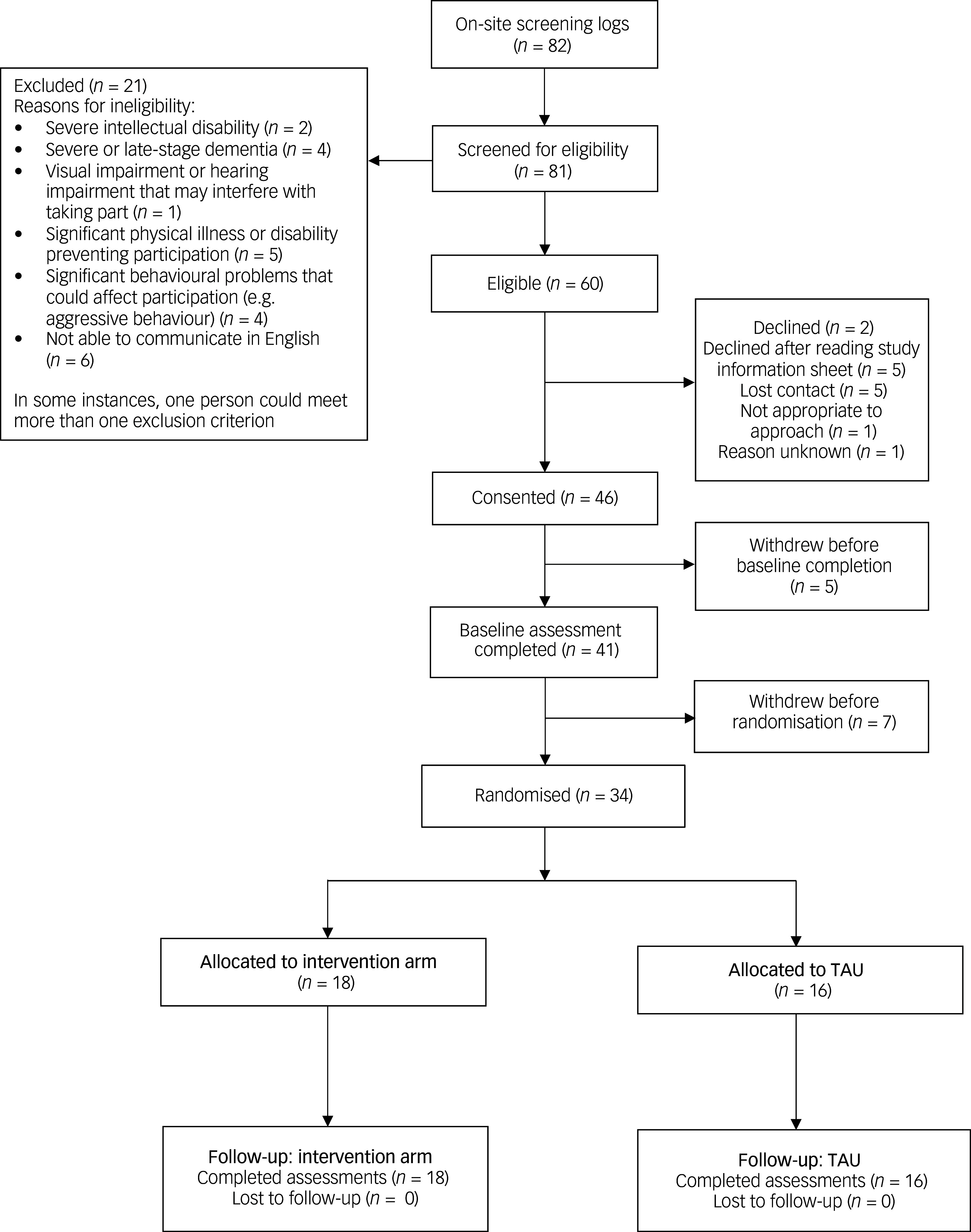
Recruitment and retention for the trial. TAU, treatment as usual.

Recruitment spanned from 1 May 2022 to 31 October 2023 (17 months); this was 5 months longer than initially planned owing to delays in opening sites and the need for multiple visits and telephone calls to enable each participant and/or their consultee to make a decision regarding participation in the study. Figure 1 shows the flow of participants through the trial. Of the 82 participants who were approached, 81 were screened and 60 were eligible. Of the 21 who were ineligible, six could not communicate in English, five had significant physical illness or disability, and 14 did not consent. Of the 60 eligible participants, 46 consented and 41 completed the baseline assessment.

Of the 41 participants who completed the baseline assessment, only 34 were randomised. Five participants withdrew before baseline completion, and seven withdrew before randomisation (12 in total). At one site, three individuals withdrew following significant delays between recruitment and the CST group, as the latter was postponed owing to a heat wave. Other reasons for withdrawal included admission to hospital (*n* = 1), death (*n* = 1), deterioration in dementia (*n* = 1), being unable to complete baseline assessment (*n* = 1), an insufficient number of participants for randomisation at one site (*n* = 2) and concerns about attending groups (*n* = 2). Of the 34 participants who were randomised, 18 were allocated to the intervention arm and 16 to TAU. There were no withdrawals post-randomisation, and all the randomised participants completed their 8–9-week follow-up.

Regarding outcome assessments, Table 3 shows the completion rates for the SIB total score, DLD, QOL-AD proxy and GDS-LD proxy outcome measures; these rates were good (less than 5% missing data) except in the case of the QOL-AD proxy, for which almost one-third of participants did not complete one item (the question about marriage, which was not applicable). Table 4 shows the scores at baseline and follow-up for the four listed outcome measures, and Table 5 shows the adjusted means. The DLD (adjusted mean −3.52; 95% CI −19.19 to 10.68) and the QOL-AD proxy (adjusted mean 1.41; 95% CI −2.44 to 5.27) showed positive changes in favour of the intervention group, whereas the results of the SIB and GDS-Proxy were in favour of the control arm. The intraclass correlation values for the GDS-LD proxy and QoL-AD proxy were negligible (<0.001), although those for the DLD (0.098) and SIB (0.29) were larger.

**Table 3:** Missing data

Outcome measures	TAU *N* = 16, *n* (%)	INT *N* = 18, *n* (%)	Total *N* = 34, *n* (%)
SIB total score			
Baseline	0 (0.0)	1 (5.6)	1 (2.9)
Follow-up	1 (6.3)	0 (0.0)	1 (2.9)
DLD			
Baseline	0 (0.0)	0 (0.0)	0 (0.0)
Follow-up	0 (0.0)	1 (5.6)	1 (2.9)
QOL-AD proxy			
Baseline	5 (31.3)	5 (27.8)	10 (29.4)
Follow-up	6 (37.5)	5 (27.8)	11 (32.4)
GDS-LD proxy			
Baseline	0 (0.0)	1 (5.6)	1 (2.9)
Follow-up	0 (0.0)	0 (0.0)	0 (0.0)

TAU, treatment as usual; INT, intervention; SIB, Severe Impairment Battery; DLD, Dementia Questionnaire for People with Learning Disabilities; QOL-AD, Quality of life in Alzheimer’s Disease; GDS-LD, Glasgow Depression Scale for People with Learning Disability.

**Table 4:** Raw scores for SIB, DLD, QOL-AD proxy and GDS-LD proxy outcome measures

Final scores of measures	Data	TAU	INT	Total
SIB total score				
Baseline	*N*	16	17	33
	Mean (s.d.)	56.50 (27.50)	62.53 (21.23)	59.61 (24.28)
	Median (IQR)	65 (45.5–75.5)	65 (46–81)	65 (46–78)
Follow-up	*N*	15	18	33
	Mean (s.d.)	59.67 (27.11)	54.94 (27.00)	57.09 (26.73)
	Median (IQR)	70 (38–79)	49.5 (41–80)	59 (41–79)
DLD sum of cognitive scores				
Baseline	*N*	16	18	34
	Mean (s.d.)	22.38 (7.74)	23.78 (9.87)	23.12 (8.83)
	Median (IQR)	22 (16.5–28)	27 (18–31)	26 (17–31)
Follow-up	*N*	16	17	33
	Mean (s.d.)	22.50 (8.51)	20.82 (10.38)	21.64 (9.41)
	Median (IQR)	23 (19.5–27)	22 (15–28)	23 (18–27)
DLD sum of social scores				
Baseline	*N*	16	18	34
	Mean (s.d.)	20.00 (7.93)	20.89 (9.89)	20.47 (8.90)
	Median (IQR)	18.5 (14.5–26.5)	20 (15–25)	19.5 (15–26)
Follow-up	*N*	16	17	33
	Mean (s.d.)	19.69 (9.19)	20.00 (12.29)	19.85 (10.73)
	Median (IQR)	19.5 (13–26)	18 (14–29)	18 (14–26)
DLD total score				
Baseline	*N*	16	18	34
	Mean (s.d.)	42.38 (15.01)	44.67 (17.24)	43.59 (16.03)
	Median (IQR)	41.5 (30–53.5)	47 (35–51)	47 (33–52)
Follow-up	*N*	16	17	33
	Mean (s.d.)	42.19 (16.23)	40.82 (20.05)	41.48 (18.03)
	Median (IQR)	42.5 (29.5–52)	44 (37–48)	44 (34–50)
QOL-AD proxy total score				
Baseline	*N*	16	18	34
	Mean (s.d.)	31.31 (4.83)	32.78 (5.79)	32.09 (5.33)
	Median (IQR)	31.5 (27–35)	33 (29–38)	32 (28–36)
Follow-up	*N*	16	18	34
	Mean (s.d.)	30.81 (5.94)	33.06 (5.65)	32.00 (5.81)
	Median (IQR)	31 (25.5–36)	34 (31–36)	33 (29–36)
GDS-LD proxy total score				
Baseline	*N*	16	18	34
	Mean (s.d.)	10.00 (3.54)	7.39 (4.37)	8.62 (4.16)
	Median (IQR)	11 (6–12.5)	7 (3–11)	8.5 (6–12)
Follow-up	*N*	16	18	34
	Mean (s.d.)	9.56 (4.97)	8.28 (5.84)	8.88 (5.40)
	Median (IQR)	11 (6–13.5)	7.5 (3–12)	8.5 (5–13)

TAU, treatment as usual; INT, intervention; SIB, Severe Impairment Battery; IQR, interquartile range; DLD, Dementia Questionnaire for People with Learning Disabilities; QOL-AD, Quality of life in Alzheimer’s Disease; GDS-LD, Glasgow Depression Scale for People with Learning Disability.

**Table 5:** Adjusted means for outcome measures from analysis of covariance

Measure	Descriptive statistics	Total	TAU	INT	Mean difference (INT − TAU)	Group favoured by results	Intraclass correlation coefficient
SIB	Adjusted mean	58.38	60.64	56.38	−4.26	TAU	0.29062
	s.e.	3.53	5.23	4.90	7.25		
	95% CI	51.10, 65.65	49.87, 71.40	46.29, 66.47	−19.19, 10.68		
DLD	Adjusted mean	41.48	43.30	39.78	−3.52	INT	0.09862
	s.e.	1.85	2.69	2.60	3.78		
	95% CI	37.68, 45.28	37.78, 48.82	34.43, 45.13	−11.29, 4.25		
GDS-LD proxy	Adjusted mean	8.88	8.47	9.27	0.80	TAU	<0.0001
	s.e.	0.81	1.20	1.16	1.72		
	95% CI	7.21, 10.54	6.00, 10.94	6.87, 11.66	−2.75, 4.34		
QoL-AD proxy	Adjusted mean	32.79	32.04	33.45	1.41	INT	<0.0001
	s.e.	0.92	1.35	1.27	1.88		
	95% CI	30.90, 34.67	29.26, 34.82	30.84, 36.07	−2.44, 5.27		

TAU, treatment as usual; INT, intervention; SIB, Severe Impairment Battery; DLD, Dementia Questionnaire for People with Learning Disabilities; QOL-AD, Quality of life in Alzheimer’s Disease; GDS-LD, Glasgow Depression Scale for People with Learning Disability.

#### CST group delivery and adherence

All the sites chose to deliver two sessions in 1 day rather than over 2 days a week. Four CST groups were delivered in total. Of these, two groups had 12 sessions delivered, one had 13 sessions and two had 14 sessions. Group sizes ranged from two to five participants. Eighteen participants were allocated to the CST arm, but two of these did not receive the intervention because there were not enough participants at one site for the group to be run. Of the possible total of 214 available sessions, 137 sessions were attended by the 16 participants, representing an attendance rate of 64.0%. Two participants who were offered CST groups did not attend any sessions, and nine (56%) attended ten or more sessions.

#### Assessment of intervention fidelity

Fidelity ratings and audio recordings were available for three of four groups. Facilitators from two groups completed the maximum of 14 ratings, and the other completed 13. Only 27 audio recordings were available (12, ten and five, respectively, from each group). The average total fidelity score rated by facilitators was 26.3 (of a possible total of 38), with a mean percentage fidelity score of 74.3%. Fifteen audio recordings were rated. The mean observer rated fidelity score was 25.4, and the percentage fidelity score was 75.9%. The agreement between facilitator and observer ratings was good at 80.6%, with an average *κ* of 0.79.

#### Acceptability of CST: qualitative findings

Twenty semi-structured interviews were conducted with nine facilitators, six carers and five participants (three males and two females, all White British, four with mild intellectual disability and one with moderate intellectual ability). The findings from the analysis of facilitator and carer interviews are presented in Table 6, summarised according to the following themes: group attendance, positive and negative experiences and impact of CST, barriers and enablers to participation and CST content. In summary, group attendance was affected by carer availability and access to transportation, as many participants had to travel far; positive benefits included social interaction and improvements in memory, communication and sleep, but participants became fatigued due to two sessions being held in 1 day. The main enablers were facilitator skill and ability to further adapt sessions and carer involvement; the main barriers were distance and travel time for participants and the amount of time needed to prepare for sessions. Findings from the content analysis of participant interviews are summarised in Table 7. Participants were largely positive about the sessions, although one participant indicated that the sessions were too long. Facilitators and carers were asked to make suggestions for improvements to the delivery and content of CST, and these are summarised in Table 8.

**Table 6:** Themes from the qualitative interviews with facilitators and carers

Theme name	Description	Examples of quotes
Group attendance	Only one group had consistent attendance. Reasons for inconsistent attendance included participants feeling anxious in the group, travel time, lack of transportation, health issues, participants feeling tired and lack of availability of carers to bring participants to groups.	‘…two participants did not turn up. One was because of the increased anxiety so couldn’t cope with this group sessions. Another participant is far from where he lives…because it’s quite far to commute…’ (Facilitator 07)
Positive and negative experiences and impact of CST	Facilitators and carers also commented that participants enjoyed engaging in activities with other group attendees. Carers noted positive changes outside the group sessions which they attributed to the group. These included better recollection of long-term memories, details about the group and general improvements in memory, communication and sleep. Facilitators commented that some members felt tired owing to two back-to-back sessions in one day and showed increased anxiety in a new setting; some became sad after a topic of discussion (e.g. difficult events in childhood). Some participants’ behaviour had a negative impact on other group members, and in one group, there was a particularly critical carer who affected the morale of the participant they were supporting. Facilitators reported experiencing frustration and wasted clinical time when participants did not attend sessions. The limited number of sessions and the ending of the group had a negative impact on one group member, as attending the group had become part of their routine.	*‘*One session where we had a snakes and ladders that we played like that was the amount of joy that was in that group was massive…that was the cherry on the cake, to be honest.’ (Facilitator 03) ‘…I’ve noticed that she’s straight away on point, responding quickly…I think it has helped with her, like memory a bit…she’s kind of gained more like communication skills really.’ (Carer 03) ‘I think when we were talking about the royal, about the queen and then she became tearful…Right. I believe that was something that reminded her of the Queen, who had passed away…’ (Facilitator 04) ‘so that was a bit frustrating because it’s quite a lot of effort for one person.’ (Facilitator 02) ‘…that one person and again towards the end of session when we talked about ending, you could see sadness because I think he really did like that peer support that consistency, he knew what was happening for the next seven weeks. So it was like in a routine structure.’ (Facilitator 01)
Barriers and enablers of participation	(a) Facilitator skill Facilitator skill in both implementing the adaptations from the CST-IDD supplement and using additional adaptations, supported group participation. Facilitators made further adaptations such as personalising sessions to participant interests, enlarging the symbols and/or pictures provided, simplifying activities (e.g. word association to picture association), reducing the number of activities (from two per session to one), swapping fine motor activities (e.g. cards) to gross motor activities (e.g. bowling), including a poster prompt with the group name, incorporating sensory activities (e.g. using play dough), using movement as part of the theme song (e.g. encouraging dancing) and moving the break to the end. (b) Relationships Promoting social interaction and connection was noted as important in setting up a positive experience. Good relationships between carers and participants were key in enabling participation but acted as a barrier when absent, especially with inconsistent attendance and facilitators not being familiar with group members and their needs before starting the group. (c) Carer support Carer support was noted to be a core enabler, with facilitators sharing that with limited carer support, e.g. to take participants to the bathroom, facilitation was difficult. Facilitators also commented on other carer support barriers, including carer staffing issues affecting consistent attendance, variable carer skill and carer disengagement from the group. (d) Distance and travel Several group members had to travel long distances, and this led to inconsistent attendance and inconvenience. Some facilitators also had to travel to different locations to deliver the group. As a result, two sessions per week were often run in the same day, creating further issues (e.g. fatigue or tiredness). (e) Group size Facilitators expressed that having fewer group participants (one to two) led to limited interactions, but too many limited one-to-one attention. (f) Preparation time Resources such as the CST-IDD supplement were more helpful than the original CST manual. However, some resources (images and physical items) were unsuitable, requiring adaptation, which could be time-consuming. (g) Session structure All groups opted to have both sessions in one day (rather than on separate days) for carer and facilitator convenience, but this was often too long for participants, resulting in boredom and fatigue. Most participants were positive about the length of sessions. Some participants confused the break with lunch and/or ending, and others lost interest after a break. Group participants appeared to engage more with the session when given choice and control (e.g. choosing the group name). Other competing demands were a barrier for some, as the group was not part of the participants’ usual routine (e.g. they had clashing social activities or were too fatigued from another activity to participate fully). Facilitators raised concerns about the site and/or venue, as some facilitators did not have full access to buildings.	‘so we had to make a lot of reasonable adjustments around simplifying that and making the activities accessible as possible.’ (Facilitator 09) ‘…because then we got social interaction amongst the three individuals which was lovely to see and it was like this is how we need to kind of try to plan… our sitting…’ (Facilitator 09) ‘…one of the biggest challenges not knowing the people who come to the group because we haven’t been able to meet them before. I think with hindsight, even just one meeting with them could have been helpful.’ (Facilitator 06) ‘…They left and then came back, so his carers weren’t there. So I think, you know, perhaps it’s helpful for the carers to hang around in the building.’ (Facilitator 02) ‘…it was over 40 minutes trip due to the traffic…He needs to use the toilet. So the transport was bit long for [name of participant].’ (Carer 04) ‘…for me, I was coming from (name of location). So a lot of clinical time was taken away…’ (Facilitator 03) ‘But when we add four, it felt a bit much and obviously when we had one well, that’s not really a group.’ (Facilitator 05) ‘…those pictures weren’t going to be any use to our clients. They were very small, not clear, so had to spend a considerable amount of time developing visuals for each session.’ (Facilitator 08) ‘…Some of the clients were getting, like quite bored in the second one…doing two on the same day and it was too much for them…’ (Facilitator 03) ‘…Then after the coffee break did not feel she wanted to stay and she keep on asking to go…’ (Facilitator 04) ‘I mean the first, because she goes to (activity Name) obviously she stopped that to come to this. So at the start we had to keep reminding her in a way to say, you know, you’re not going to (activity name), you’re going to the CST…’ (Carer 03)
CST content	Some general barriers were conversational tasks being based too heavily on verbal communication, and ‘sit down’ activities and tasks which were ‘too easy’ or ‘too difficult’ for participants. Tasks which required word skills were often deemed too complex (e.g. word association) unless adapted to a simpler version (e.g. picture association) as suggested by the CST-IDD supplement. Enablers were active games that did not require fine motor or word skills (e.g. bowling, throwing beanbags). Participants enjoyed sessions with tangible resources, including the music and childhood sessions. The money session worked for some and not others, highlighting the variability in daily living skills. The food session raised some risks with using real food (e.g. dysphagia), and some play food was provided in the box of resources. However, some of the labelling was too small or play food was too abstract for some participants.	

CST, cognitive stimulation therapy; CST-IDD, CST for people with intellectual disability and dementia.

**Table 7:** Basic content analysis of participant interviews

Content analysis categories	Description	Quote
Pre-interview session and/or activity	All participants had a positive experience of the pre-interview activity (money, bingo, word games and bowling).	
Being in a group	Participants liked being in a group with carer and facilitator support.	‘It’s been you know really good with meet…meeting different people.’ (P03)
Session length	Three participants liked the length of the sessions, one was unsure and one thought that it was too long.	‘it’s is…long.’ (P04)
Number of sessions per week	Four participants liked the number of sessions, but one participant wanted more sessions.	‘If if I could come in more more often.’ (P03)
Breaks in the middle of the session	All participants indicated that they liked having breaks.	
Group song	All participants liked the group song.	‘I like humming [name of tune].’ (P05)
Talking and hearing about other members’ experiences	All participants liked hearing other members share experiences. One participant revealed that they did not get on with another member.	‘I I do like people talking about different things.’ (P03) ‘Well, that (name) girl, she’s…she’s alright but she goes…she does my head in.’ (P02)
Comments and/or suggestions	One participant was enthusiastic about their friend joining.	‘if we could… if we could get a couple, probably not now but when we have the next session could we have some new people join us?… cause I’ve got another guy with this in the same house as me…He would love to come along.’ (P03)

P, participant.

**Table 8:** Summary of recommendations for future groups from facilitator and carer interviews

Group delivery and set-up	
Personalisation	Personalised to participant interests
Set-up	Meeting participants and carers before the first session to gather key information (e.g. personal interests)
	Meeting participants between sessions to improve confidence and familiarity
	Pre-adapted resources
	Planning the break in more detail
	Plan to continue activities at home if beneficial
	Single-borough groups only (minimises unmanaged risk)
	Plan sessions in detail (e.g. check access to resources)
	Thorough risk assessment and planning
	Specific inclusion criteria (mild intellectual disability and dementia) or a similar level of functioning per group
Practical considerations	
Carer support	Remain nearby to support
Distance and travel	Convenient location for all
	Transport options
External environment	Mid-morning session
	No extreme temperatures
Session and day structure	Shorter sessions (not 2 h)
Site and/or venue	Check room suitability
Cognitive stimulation therapy content	
Activities/sessions	Active games rather than sit-down activities
	Creative activities
	No fine motor activities
	Larger games (increased accessibility)
	Appropriate activities for available provision (check food safety, kitchen requirements)
	Less based on intact communication and more sensory driven (arts, painting)

#### Adverse events

There were six serious adverse events during the trial (two deaths and four hospital admissions), which were all unrelated to trial participation.

### Health economics data completeness results

Analysis of health economic data was conducted using StataNow release 18.5 for Windows. All 18 participants in the TAU arm and 15 of 16 participants in the CST arm completed the health-related quality of life questionnaires that were administered to them. The EQ-5D-5L proxy questionnaire completed by carers had no missing items, whereas there were a few missing items for the self-reported measures: EQ-5D-5L (up to three missing items per arm) and the new modified EQ-5D-3L for adults with intellectual disability (up to five missing items per arm). As the sample sizes were small, no conclusions could be drawn regarding the different levels of completion of the different measures. The item responses, including the visual analogue scale score, and the calculated utility scores can be found in Supplementary Tables 1–3 available at https://doi.org/10.1192/bjo.2025.10764. In the modified CSRI, most participants reported some general practitioner visits, and smaller numbers reported seeing a psychiatrist, a learning disabilities nurse or professionals from the other categories that were listed in the questionnaire (Supplementary Tables 4 and 5). Information on reported use of prescription and over-the-counter medications is given in Supplementary Tables 6 and 7.

### Cost of CST-IDD group sessions

The total cost of running the sessions, including staff time for training, staff time for delivering CST, manual purchase and consumables costs, was calculated as the sum of these items (see Tables 9 and 10 and Supplementary Table 8). The total was calculated to be £9636 across all groups and participants, corresponding to £2409 per group (four groups) and £602 per participant (16 participants).

**Table 9:** Costs of delivering cognitive stimulation therapy

Type of cost	Explanation/comment
Cost of 1-day course for training facilitators to use the manual (including time spent by trainer and by facilitators)	Trainer: 1 day (6 h) of consultant psychiatrist, band 8c Facilitators: 1 day (6 h) for each facilitator being trained, band 5 Additional training (1.5 h) on the manual supplement: band 5 trainer time plus facilitators’ time
Staff time for facilitators running the session	There were one or two facilitators at each session across the four sites, meaning an average of 1.5 facilitators per session (band 5) Each session lasted for 1 h Preparatory work before sessions was estimated at 1 h per facilitator per session
Purchase of one copy of the manual per centre	Cost £18 per copy, four copies in total
Cost of resources for the group sessions – for a breakdown, see Supplementary Table 8	Not including room booking costs and refreshments; not including travel costs for participants or carers

**Table 10:** Intervention cost, calculated as the sum of staff time spent on training and on session preparation and delivery, and the expenditure on resources for the sessions (from Supplementary Table 8)

	Time (h)	Hourly rate (£)	Number of people	Total cost	Source (for hourly rate)
Training on manual					
Trainer (band 8c)	6	118	1	£708	PSSRU 2023 Table 8.2^28^
Facilitators (band 5)	6	41	6	£1476	PSSRU 2023 Table 8.2
Training on supplement					
Trainer (band 5)	1.5	41	1	£62	PSSRU 2023 Table 8.2
Facilitators (band 5)	1.5	41	6	£369	PSSRU 2023 Table 8.2
	Time (h)	Hourly rate (£)	Facilitators per session	Total cost	Source (for hourly rate)
Running the sessions					
Preparation (53 × 1 h)	53	41	1.5	£3260	PSSRU 2023 Table 8.2
Delivery (53 × 1 h)	53	41	1.5	£3260	PSSRU 2023 Table 8.2
					Source
Resources for sessions, including purchase of manual				£503	See Supplementary Table 8
	Total (all participants)			£9636	
	Total (per participant)			£602	

PSSRU, Personal Social Services Research Unit.

### Progression criteria

Table 11 summarises the feasibility outcomes and the extent to which the progression criteria were met. The only feasibility outcomes to meet the ‘Go’ criteria were recruitment (adequate recruitment, and recruitment rate), percentage of sessions delivered by facilitators and serious adverse events. All the other outcomes, including retention, group attendance and acceptability, were in the ‘Review’ category.

**Table 11:** Summary of the feasibility outcomes and whether they met progression criteria

Feasibility outcome	Description	Progression criteria met (Go/Review/Stop)	Comments
Recruitment of participants	Adequate recruitment	46 participants recruited: Go	Only 34 were actually randomised, so Review status might be more appropriate
	Eligibility rate	81 screened; 60 eligible (74.1%): Review	
	Consent/recruitment rate	46 recruited from 60 eligible people (76.7%): Go	
Retention of participants in the study		46 recruited but only 34 completed trial (73.9%): Review	All drop-outs occurred before randomisation
Suitability of outcome measures	Completion rates of outcome measures	<5% missing data for most of the measures apart from QOL-AD proxy: Go.	One item on QOL-AD proxy was not applicable for most participants
Adherence	Number of sessions attended by participants	56% of participants attended ten or more sessions: Review	
	Percentage of sessions delivered by facilitators	53 of 56 sessions were delivered (95%): Go	One site did not run a group owing to too few participants, meaning 75.7% of sessions were actually delivered (53/70); Review
Fidelity	Extent to which intervention components were delivered (percentage score)	Moderate fidelity (74.3 to 75.9%) based on observer and self-ratings by facilitators: Review	
Acceptability	Satisfaction with CST	Stakeholders identified positive experiences and benefits from CST but some barriers were highlighted: Review	
Adverse events		No serious adverse events: Go	

CST, cognitive stimulation therapy; QOL-AD, Quality of life in Alzheimer’s Disease.

## Discussion

### Summary of findings

This was the first feasibility RCT of group CST in people with intellectual disability and dementia. We achieved the ‘Go’ criteria for recruitment (total number of participants recruited and consent/recruitment rate). However, a longer period of recruitment than planned was needed (17 months instead of 12 months), and 12 participants dropped out following consent, with only 34 randomised. Reasons included one group having to be postponed owing to an extreme heat wave. There were often several months between participants providing informed consent and the baseline assessment being conducted, as groups could not go ahead until the minimum number of participants had been recruited. The large attrition rate between consent and randomisation affected the retention rate, which was just below our target of 75% (‘Review’ category).

There was an indication that the results from the DLD and QOL-AD proxy favoured the intervention group, whereas those from other measures, the SIB and GDS proxy, favoured the control group; however, none of these differences was significant. These results need to be interpreted with caution given the small sample size. However, all measures appeared to be appropriate for this population.

Although most of the sessions were delivered (‘Go’), group attendance was poor, with only 56% of participants attending ten or more sessions (‘Review’). Factors affecting group attendance, as highlighted by the qualitative interviews, included lack of carer and transport availability, location and travelling distance, and groups clashing with other commitments. However, one group reported very good attendance. The strategies used by facilitators to promote attendance included calling carers and participants to remind them about the session and helping to resolve issues with transport. Other factors that contributed to feasibility outcomes such as group attendance included facilitator skill, appropriate adaptations and social interactions, which fostered positive engagement in sessions.

The fidelity of delivery of CST was of moderate quality (‘Review’). The interviews with participants, carers and facilitators highlighted positive experiences such as enjoyment of activities and sharing experiences with other group members, as well as benefits of CST with respect to participants’ memory, communication and sleep. Overall, the groups were perceived positively by participants, albeit with some barriers including fatigue due to two sessions on 1 day and the need to adapt and modify CST sessions further.

The healthcare and social care resource use questionnaires were well completed. The proxy version of the EQ-5D yielded the highest completion rates of the three questionnaires, although the sample sizes for both self-completed versions were small and therefore no meaningful conclusions could be drawn regarding completion rates. The average cost of CST was £602 per participant, which was somewhat higher than the cost for people with dementia without intellectual disability.^29^ The higher cost was largely due to the smaller group sizes.

### Results in context

The results of this study suggest that group CST was associated with small improvements in cognition and quality of life (although the study was not powered to detect a significant difference); this was consistent with CST studies in people with dementia without intellectual disability, which have found improvements in cognition and quality of life.^8^ Our previous feasibility RCT of individual CST found improvements in quality of life but not cognition.^10^ The views of participants regarding being in the CST group were largely positive, and their carers reported positive benefits outside the groups, similar to the findings of studies of CST in people with dementia without intellectual disability.^30,31^ Other themes that emerged, such as the importance of carer engagement (e.g. for transport) and facilitators having appropriate skills and the time and resources needed for preparation, were shared across a number of studies.^31^ Fidelity was particularly poor in our study of individual CST, which was delivered by family and paid carers; fidelity in the present study was of moderate quality, indicating that professionals are more likely to deliver the intervention as intended, which might in turn lead to improved outcomes.

### Strengths and limitations

One of the strengths of the study was the inclusion of the voices of people with dementia and intellectual disability about their experiences of CST groups, something that is often lost within research processes.^7^ This was a multicentre study across six NHS sites in England and included sites from both urban and more rural areas. The inclusion of such diverse sites provided information about potential barriers relating to group attendance, which was one of the key issues highlighted in the study.

Despite the study taking place in ethnically diverse regions of England, the vast majority of participants with intellectual disability were from White ethnic backgrounds. This may reflect broader issues such as delayed diagnosis of dementia in people from minority ethnic groups,^32^ but there needs to be a concerted effort to include these groups in dementia research. Another issue encountered in this study was the heterogeneity among group participants, with groups comprising individuals with both mild and moderate intellectual disability and dementia. It is preferable to have CST groups of individuals with similar abilities, as this promotes better engagement and inclusion. It was not possible to run separate groups based on abilities owing to issues with recruitment and not having a sufficient number of participants at each site. It would have been preferable to have delivered CST groups at participants’ usual day services such as day centres, as is the case for CST studies in people with dementia without intellectual disability, and this may have reduced burden and transportation costs.

To estimate the intervention cost, we did not include costs for room bookings or refreshments, as these were not recorded. We also did not include costs to participants, families and/or carers such as transport costs, and we did not include time spent by paid carers who facilitated participants’ attendance, although with hindsight we would recommend that this latter cost in particular should be included in a future full trial. Therefore, the overall cost of CST per participant is likely to be higher. We also did not include follow-up assessments beyond the end of the intervention period, nor did we collect data on the cause of intellectual disability (e.g. Down syndrome), although many of the participants did have Down syndrome, owing to its association with dementia.

### Recommendations and implications

A future study would need to consider strategies to mitigate the issues highlighted in this study. Recruitment was affected by the lack of eligible participants. At one site, this was because of a long waiting list for dementia assessments. In addition, dementia diagnoses for people with intellectual disability usually require longitudinal assessments,^32^ which can result in diagnosis at later stages of the condition, when the individual may no longer be eligible for CST. In our study, almost all the participants had either Alzheimer’s dementia, vascular dementia or a mixed dementia. This was probably because of the strong association between Down syndrome and Alzheimer’s dementia, but it may also reflect challenges in diagnosing other types of dementia in this population. It is crucial that the diagnostic pathway is improved, and it may be necessary to include additional resources to support dementia assessments in any future research.

There were some difficulties around attendance, and any future research should consider strategies to make groups more accessible for participants and their carers. This might include careful consideration of the location (e.g. a day centre) and allocation of a budget to support participants who may not be able to afford transport or to pay for carers’ time. A future study also could explore the feasibility of attending an online CST group. Feasibility studies of people with dementia without intellectual disability, across a number of countries, have found online delivery to be acceptable, although data on effectiveness are not yet available.^33^ Remote delivery of CST groups will have specific challenges for people with intellectual disability and dementia, but it may improve access and attendance.

The CST supplement was found to be helpful in providing suggestions for modifying activities. However, facilitators commented on the need for further adaptations based on the ability of participants and for challenges with engagement to be addressed; some of the CST content and resources in the supplement were not suitable. In light of these comments, a further revision of the supplement is warranted. The supplement and training should also include guidance on modifications to the session format, such as shorter sessions incorporating more breaks, varied group sizes depending on abilities, and how to work with and manage carers who are present during the groups. A longer training session and supervision of facilitators (e.g. every 2 weeks) might be helpful in addressing and overcoming some of the challenges in delivering the intervention.

The DLD and the QOL-AD proxy showed some sensitivity to change in favour of the intervention, in line with findings of other CST studies, indicating that they were appropriate measures to assess treatment effect within this population. There is no evidence to distinguish between the appropriateness of the EQ-5D-5L proxy or modified EQ-5D-3L; therefore, we would recommend using both in a potential future main trial.

The sample size calculation for a definitive trial will need to include approximately 25% attrition owing to the high observed rate of withdrawal between consent and randomisation, as well as accommodating expected attrition in a larger sample to follow-up. The sample size calculation would also need to account for clustering in the intervention group by considering an appropriate design effect.

Overall, this RCT of group CST in people with intellectual disability and dementia provides valuable information about the feasibility and acceptability of a future definitive trial. On the basis of our findings, it may only be possible to conduct a future RCT if there are improvements to the dementia diagnostic pathway within clinical services, coupled with considerable revision of trial processes and procedures such as the recruitment strategy, reductions in delays between consent and baseline assessments, and strategies to improve group attendance.

## Supporting information

Ali et al. supplementary material Ali et al. supplementary material

## Data Availability

The data that support the findings of this study are available from the corresponding author, A.A., upon reasonable request.

## Supplementary material

The supplementary material is available online at https://doi.org/10.1192/bjo.2025.10764
