# Supplementary material for: Group cognitive stimulation therapy for people with intellectual disability and dementia: feasibility randomised controlled trial
Source: BJPsych Open. 2025 Aug 1;11(5):e168. doi: 10.1192/bjo.2025.10764 (PMC12344426; doi:10.1192/bjo.2025.10764)
Supplement: Ali et al. supplementary material [file S2056472425107643sup001.docx]

**Supplementary Tables**

**Table S1: Raw scores of EQ-5D measures – individual items and utility scores calculated from the 5 dimensions in each case – all participants both arms**

| **TOTAL (both arms)** | **Baseline** |  |  | **Follow-up** |  |  |
| --- | --- | --- | --- | --- | --- | --- |
|  | **N** | **Mean (SD)** | **Median [IQR]** | **N** | **Mean (SD)** | **Median [IQR]** |
| **EQ-5D-5L proxy** |  |  |  |  |  |  |
| Mobility | 33 | 2.5 (1.3) | 2 [1 - 3] | 33 | 2.3 (1.3) | 2 [1 - 3] |
| Self-care | 33 | 3.1 (1.2) | 3 [2 - 4] | 33 | 3.1 (1.4) | 3 [2 - 4] |
| Usual activities | 33 | 2.4 (1.3) | 2 [1 - 3] | 33 | 2.2 (1.2) | 2 [1 - 3] |
| Pain / discomfort | 33 | 1.8 (0.9) | 2 [1 - 3] | 33 | 1.6 (0.8) | 1 [1 - 2] |
| Anxiety / depression | 33 | 1.9 (0.8) | 2 [1 - 2] | 33 | 1.7 (0.9) | 1 [1 - 2] |
| VAS (scale 0 to 100) | 33 | 63.8 (15.7) | 60 [50 - 75] | 32 | 68.4 (17.2) | 70 [57.5 - 80] |
| Utility score (VSE tariff) | 33 | 0.7 (0.2) | 0.7 [0.6 - 0.8] | 33 | 0.7 (0.2) | 0.75 [0.56 - 0.87] |
| Utility score (mapping) | 33 | 0.5 (0.3) | 0.6 [0.4 - 0.7] | 33 | 0.6 (0.3) | 0.64 [0.46 - 0.79] |
| **EQ-5D-5L self complete** |  |  |  |  |  |  |
| Mobility | 15 | 1.1 (0.3) | 1 [1 - 1] | 16 | 1.3 (0.6) | 1 [1 - 1.5] |
| Self-care | 15 | 1 (0) | 1 [1 - 1] | 15 | 1.2 (0.6) | 1 [1 - 1] |
| Usual activities | 15 | 1 (0) | 1 [1 - 1] | 16 | 1.3 (0.6) | 1 [1 - 1] |
| Pain / discomfort | 15 | 1.5 (0.8) | 1 [1 - 2] | 16 | 1.4 (0.8) | 1 [1 - 1.5] |
| Anxiety / depression | 15 | 1.2 (0.4) | 1 [1 - 1] | 16 | 1.3 (0.5) | 1 [1 - 2] |
| VAS (scale 0 to 100) | 14 | 87.1 (12.5) | 90 [80 - 95] | 15 | 80 (17) | 85 [70 - 95] |
| Utility score (VSE tariff) | 15 | 0.9 (0.1) | 1 [0.9 - 1] | 15 | 0.9 (0.1) | 1 [0.86 - 1] |
| Utility score (mapping) | 15 | 0.9 (0.1) | 1 [0.8 - 1] | 15 | 0.9 (0.1) | 0.97 [0.71 - 0.98] |
| **EQ-5D-3L modified** |  |  |  |  |  |  |
| Mobility | 11 | 1.6 (0.9) | 1 [1 - 3] | 13 | 1.6 (0.8) | 1 [1 - 2] |
| Self-care | 10 | 2.6 (0.7) | 3 [2 - 3] | 13 | 2.4 (0.7) | 2 [2 - 3] |
| Usual activities | 10 | 1.2 (0.4) | 1 [1 - 1] | 11 | 1.5 (0.8) | 1 [1 - 2] |
| Pain / discomfort | 12 | 1.4 (0.8) | 1 [1 - 1.5] | 12 | 1.8 (0.8) | 2 [1 - 2.5] |
| Anxiety / depression | 12 | 1.3 (0.5) | 1 [1 - 2] | 11 | 1.2 (0.4) | 1 [1 - 1] |
| VAS (scale 0 to 10) | 11 | 7.6 (2.9) | 9 [5 - 10] | 11 | 8 (3.1) | 10 [6 - 10] |
| Utility score (3L tariff) | 10 | 0.3 (0.3) | 0.2 [0.1 - 0.4] | 11 | 0.3 (0.3) | 0.26 [0.11 - 0.82] |

SD = standard deviation, IQR = interquartile range, VSE = Value Set for England, N = sample size

**Table S2: Raw scores of EQ-5D measures – individual items and utility scores calculated from the 5 dimensions in each case – TAU arm**

| **TAU** | **Baseline** |  |  | **Follow-up** |  |  |
| --- | --- | --- | --- | --- | --- | --- |
|  | **N** | **Mean (SD)** | **Median [IQR]** | **N** | **Mean (SD)** | **Median [IQR]** |
| **EQ-5D-5L proxy** |  |  |  |  |  |  |
| Mobility | 16 | 2.4 (1.4) | 2 [1 - 3.5] | 16 | 2.3 (1.4) | 2 [1 - 3.5] |
| Self-care | 16 | 3.3 (1.1) | 3 [2.5 - 4] | 16 | 3.1 (1.4) | 3 [2 - 4.5] |
| Usual activities | 16 | 2.4 (1.3) | 2 [1.5 - 3] | 16 | 2.3 (1.2) | 2 [1 - 3] |
| Pain / discomfort | 16 | 1.8 (1) | 1.5 [1 - 2.5] | 16 | 1.5 (0.9) | 1 [1 - 2] |
| Anxiety / depression | 16 | 2.1 (0.8) | 2 [1.5 - 3] | 16 | 1.7 (0.9) | 1 [1 - 2.5] |
| VAS (scale 0 to 100) | 16 | 63.4 (13.4) | 60 [52.5 - 77.5] | 15 | 60.3 (13.3) | 60 [50 - 70] |
| Utility score (VSE tariff) | 16 | 0.6 (0.2) | 0.7 [0.5 - 0.8] | 16 | 0.7 (0.2) | 0.71 [0.56 - 0.87] |
| Utility score (mapping) | 16 | 0.5 (0.2) | 0.6 [0.4 - 0.7] | 16 | 0.6 (0.3) | 0.62 [0.46 - 0.81] |
| **EQ-5D-5L self complete** |  |  |  |  |  |  |
| Mobility | 8 | 1.1 (0.4) | 1 [1 - 1] | 8 | 1.3 (0.7) | 1 [1 - 1] |
| Self-care | 8 | 1 (0) | 1 [1 - 1] | 7 | 1.3 (0.8) | 1 [1 - 1] |
| Usual activities | 8 | 1 (0) | 1 [1 - 1] | 8 | 1.4 (0.7) | 1 [1 - 1.5] |
| Pain / discomfort | 8 | 1.6 (1.1) | 1 [1 - 2] | 8 | 1.5 (0.9) | 1 [1 - 2] |
| Anxiety / depression | 8 | 1.1 (0.4) | 1 [1 - 1] | 8 | 1.3 (0.5) | 1 [1 - 1.5] |
| VAS (scale 0 to 100) | 7 | 89.3 (15.7) | 95 [90 - 100] | 7 | 75.7 (17.9) | 75 [60 - 95] |
| Utility score (VSE tariff) | 8 | 0.9 (0.1) | 1 [0.9 - 1] | 7 | 0.9 (0.1) | 1 [0.84 - 1] |
| Utility score (mapping) | 8 | 0.9 (0.1) | 0.9 [0.8 - 1] | 7 | 0.9 (0.2) | 0.98 [0.69 - 0.98] |
| **EQ-5D-3L modified** |  |  |  |  |  |  |
| Mobility | 5 | 1.8 (1.1) | 1 [1 - 3] | 6 | 1.8 (0.8) | 2 [1 - 2] |
| Self-care | 5 | 2.6 (0.9) | 3 [3 - 3] | 6 | 2.2 (0.8) | 2 [2 - 3] |
| Usual activities | 5 | 1.2 (0.4) | 1 [1 - 1] | 5 | 2 (1) | 2 [1 - 3] |
| Pain / discomfort | 5 | 1.6 (0.9) | 1 [1 - 2] | 5 | 2.2 (0.8) | 2 [2 - 3] |
| Anxiety / depression | 5 | 1 (0) | 1 [1 - 1] | 5 | 1.4 (0.5) | 1 [1 - 2] |
| VAS (scale 0 to 10) | 4 | 8 (2.8) | 9 [6 - 10] | 5 | 6.2 (3.8) | 6 [3 - 10] |
| Utility score calculated using 3L tariff | 5 | 0.3 (0.3) | 0.1 [0.1 - 0.4] | 5 | 0.1 (0.2) | 0.2 [0.11 - 0.26] |

SD = standard deviation, IQR = interquartile range, VSE = Value Set for England, N = sample size

**Table S3: Raw scores of EQ-5D measures – individual items and utility scores calculated from the 5 dimensions in each case – CST arm**

| **CST arm** | **Baseline** |  |  | **Follow-up** |  |  |
| --- | --- | --- | --- | --- | --- | --- |
|  | **N** | **Mean (SD)** | **Median [IQR]** | **N** | **Mean (SD)** | **Median [IQR]** |
| **EQ-5D-5L proxy** |  |  |  |  |  |  |
| Mobility | 17 | 2.5 (1.3) | 2 [2 - 3] | 17 | 2.4 (1.3) | 2 [1 - 3] |
| Self-care | 17 | 2.8 (1.3) | 2 [2 - 4] | 17 | 3 (1.5) | 3 [2 - 4] |
| Usual activities | 17 | 2.4 (1.5) | 2 [1 - 3] | 17 | 2.1 (1.3) | 2 [1 - 3] |
| Pain / discomfort | 17 | 1.9 (0.9) | 2 [1 - 3] | 17 | 1.7 (0.8) | 2 [1 - 2] |
| Anxiety / depression | 17 | 1.8 (0.8) | 2 [1 - 2] | 17 | 1.7 (1) | 1 [1 - 2] |
| VAS (scale 0 to 100) | 17 | 64.1 (18) | 70 [50 - 75] | 17 | 75.6 (17.5) | 75 [70 - 80] |
| Utility score (VSE tariff) | 17 | 0.7 (0.2) | 0.7 [0.6 - 0.9] | 17 | 0.7 (0.3) | 0.8 [0.56 - 0.87] |
| Utility score (mapping) | 17 | 0.6 (0.3) | 0.6 [0.4 - 0.8] | 17 | 0.6 (0.3) | 0.68 [0.39 - 0.79] |
| **EQ-5D-5L self complete** |  |  |  |  |  |  |
| Mobility | 7 | 1 (0) | 1 [1 - 1] | 8 | 1.4 (0.5) | 1 [1 - 2] |
| Self-care | 7 | 1 (0) | 1 [1 - 1] | 8 | 1.1 (0.4) | 1 [1 - 1] |
| Usual activities | 7 | 1 (0) | 1 [1 - 1] | 8 | 1.1 (0.4) | 1 [1 - 1] |
| Pain / discomfort | 7 | 1.3 (0.5) | 1 [1 - 2] | 8 | 1.4 (0.7) | 1 [1 - 1.5] |
| Anxiety / depression | 7 | 1.3 (0.5) | 1 [1 - 2] | 8 | 1.4 (0.5) | 1 [1 - 2] |
| VAS (scale 0 to 100) | 7 | 85 (9.1) | 85 [80 - 95] | 8 | 83.8 (16.4) | 87.5 [77.5 - 95] |
| Utility score (VSE tariff) | 7 | 1 (0.1) | 1 [0.9 - 1] | 8 | 0.9 (0.1) | 0.93 [0.89 - 1] |
| Utility score (mapping) | 7 | 0.9 (0.1) | 1 [0.8 - 1] | 8 | 0.9 (0.1) | 0.88 [0.79 - 0.98] |
| **EQ-5D-3L modified** |  |  |  |  |  |  |
| Mobility | 6 | 1.5 (0.8) | 1 [1 - 2] | 7 | 1.4 (0.8) | 1 [1 - 2] |
| Self-care | 5 | 2.6 (0.5) | 3 [2 - 3] | 7 | 2.6 (0.5) | 3 [2 - 3] |
| Usual activities | 5 | 1.2 (0.4) | 1 [1 - 1] | 6 | 1.2 (0.4) | 1 [1 - 1] |
| Pain / discomfort | 7 | 1.3 (0.8) | 1 [1 - 1] | 7 | 1.6 (0.8) | 1 [1 - 2] |
| Anxiety / depression | 7 | 1.6 (0.5) | 2 [1 - 2] | 6 | 1 (0) | 1 [1 - 1] |
| VAS (scale 0 to 10) | 7 | 7.4 (3.2) | 9 [5 - 10] | 6 | 9.5 (1.2) | 10 [10 - 10] |
| Utility score calculated using 3L tariff | 5 | 0.3 (0.3) | 0.4 [0.1 - 0.4] | 6 | 0.5 (0.3) | 0.63 [0.24 - 0.82] |

SD = standard deviation, IQR = interquartile range, VSE = Value Set for England, N = sample size

| **Table S4: Primary and community health care resource use reported for individual CSRI items – all participants in both arms** | | | | | | | | |
| --- | --- | --- | --- | --- | --- | --- | --- | --- |
|  | | | | | | | | |
| **TOTAL (both arms)** | **Baseline** |  |  |  | **Follow-up** |  |  |  |
|  | **Number of participants reporting non-zero use** | **Mean visits (SD)** | **Median visits [IQR]** | **How many private?** | **Number of participants reporting non-zero use** | **Mean visits (SD)** | **Median visits [IQR]** | **How many private?** |
| **GP** | **20** |  |  | **1** | **13** |  |  | **1** |
| GP clinic | 16 | 1.3 (0.8) | 1 [1 - 1.5] |  | 11 | 1.3 (0.8) | 1 [1 - 2] |  |
| GP video | 9 | 0 (0) | 0 [0 - 0] |  | 7 | 0.3 (0.8) | 0 [0 - 0] |  |
| GP phone | 9 | 0.1 (0.3) | 0 [0 - 0] |  | 7 | 0.4 (0.5) | 0 [0 - 1] |  |
| GP home | 13 | 0.5 (0.9) | 0 [0 - 1] |  | 7 | 0.1 (0.4) | 0 [0 - 0] |  |
| **Community nurse (CN)** | **5** |  |  | **0** | **2** |  |  | **0** |
| CN clinic | 2 | 4.5 (4.9) | 4.5 [1 - 8] |  | 1 | 1 (0) | 1 [1 - 1] |  |
| CN video | 1 | 0 (0) | 0 [0 - 0] |  | 1 | 0 (0) | 0 [0 - 0] |  |
| CN phone | 1 | 0 (0) | 0 [0 - 0] |  | 1 | 0 (0) | 0 [0 - 0] |  |
| CN home | 4 | 1.3 (1) | 1.5 [0.5 - 2] |  | 1 | 0 (0) | 0 [0 - 0] |  |
| **Community Psychiatric Nurse (CPN)** | **2** |  |  | **0** | **2** |  |  | **0** |
| CPN clinic | 2 | 1 (0) | 1 [1 - 1] |  | 2 | 0 (0) | 0 [0 - 0] |  |
| CPN video | 2 | 0 (0) | 0 [0 - 0] |  | 2 | 0 (0) | 0 [0 - 0] |  |
| CPN phone | 2 | 0 (0) | 0 [0 - 0] |  | 2 | 0.5 (0.7) | 0.5 [0 - 1] |  |
| CPN home | 2 | 0 (0) | 0 [0 - 0] |  | 2 | 0.5 (0.7) | 0.5 [0 - 1] |  |
| **Learning Disabilities Nurse (LDN)** | **8** |  |  | **0** | **8** |  |  | **0** |
| LDN clinic | 3 | 1 (0) | 1 [1 - 1] |  | 7 | 0.9 (0.4) | 1 [1 - 1] |  |
| LDN video | 4 | 0.3 (0.5) | 0 [0 - 0.5] |  | 4 | 0 (0) | 0 [0 - 0] |  |
| LDN phone | 5 | 2.6 (3.3) | 2 [0 - 3] |  | 5 | 0.6 (0.5) | 1 [0 - 1] |  |
| LDN home | 6 | 0.7 (0.8) | 0.5 [0 - 1] |  | 4 | 0 (0) | 0 [0 - 0] |  |
| **Health facilitator (HF)** | **0** |  |  | **0** | **0** |  |  | **0** |
| HF clinic |  |  |  |  |  |  |  |  |
| HF video |  |  |  |  |  |  |  |  |
| HF phone |  |  |  |  |  |  |  |  |
| HF home |  |  |  |  |  |  |  |  |
| **Care manager/social worker (CMSW)** | **3** |  |  | **0** | **4** |  |  | **0** |
| CMSW clinic | 0 | 0 (0) | 0 [0 - 0] |  | 3 | 0.3 (0.6) | 0 [0 - 1] |  |
| CMSW video | 0 | 0 (0) | 0 [0 - 0] |  | 3 | 0.3 (0.6) | 0 [0 - 1] |  |
| CMSW phone | 1 | 4 (0) | 4 [4 - 4] |  | 3 | 0.7 (1.2) | 0 [0 - 2] |  |
| CMSW home | 3 | 1.7 (0.6) | 2 [1 - 2] |  | 4 | 0.5 (0.6) | 0.5 [0 - 1] |  |
| **Advocate** | **1** |  |  | **0** | **2** |  |  | **0** |
| Advocate clinic | 0 | 0 (0) | 0 [0 - 0] |  | 2 | 1 (0) | 1 [1 - 1] |  |
| Advocate video | 0 | 0 (0) | 0 [0 - 0] |  | 1 | 0 (0) | 0 [0 - 0] |  |
| Advocate phone | 1 | 3 (0) | 3 [3 - 3] |  | 1 | 0 (0) | 0 [0 - 0] |  |
| Advocate home | 1 | 3 (0) | 3 [3 - 3] |  | 1 | 0 (0) | 0 [0 - 0] |  |
| **Community support worker (CSW)** | **1** |  |  | **0** | **2** |  |  | **0** |
| CSW clinic | 0 | 0 (0) | 0 [0 - 0] |  | 1 | 0 (0) | 0 [0 - 0] |  |
| CSW video | 0 | 0 (0) | 0 [0 - 0] |  | 1 | 0 (0) | 0 [0 - 0] |  |
| CSW phone | 0 | 0 (0) | 0 [0 - 0] |  | 2 | 0.5 (0.7) | 0.5 [0 - 1] |  |
| CSW home | 1 | 8 (0) | 8 [8 - 8] |  | 2 | 1 (0) | 1 [1 - 1] |  |
| **NHS Direct or call '111'** | **3** |  |  | **0** | **5** |  |  | **0** |
| NHS Direct clinic | 2 | 0 (0) | 0 [0 - 0] |  | 4 | 0.8 (0.5) | 1 [0.5 - 1] |  |
| NHS Direct video | 2 | 0 (0) | 0 [0 - 0] |  | 4 | 0 (0) | 0 [0 - 0] |  |
| NHS Direct phone | 3 | 1.3 (0.6) | 1 [1 - 2] |  | 5 | 0.4 (0.9) | 0 [0 - 0] |  |
| NHS Direct home | 2 | 0 (0) | 0 [0 - 0] |  | 4 | 0.3 (0.5) | 0 [0 - 0.5] |  |
| **Psychotherapy (IAPT)** | **0** |  |  | **0** | **0** |  |  | **0** |
| Psychotherapy clinic |  |  |  |  |  |  |  |  |
| Psychotherapy video |  |  |  |  |  |  |  |  |
| Psychotherapy phone |  |  |  |  |  |  |  |  |
| Psychotherapy home |  |  |  |  |  |  |  |  |
| **Psychiatrist** | **17** |  |  | **0** | **6** |  |  | **0** |
| Psychiatrist clinic | 14 | 0.9 (0.3) | 1 [1 - 1] |  | 6 | 0.8 (0.4) | 1 [1 - 1] |  |
| Psychiatrist video | 9 | 0 (0) | 0 [0 - 0] |  | 2 | 0.5 (0.7) | 0.5 [0 - 1] |  |
| Psychiatrist phone | 9 | 0.1 (0.3) | 0 [0 - 0] |  | 2 | 0 (0) | 0 [0 - 0] |  |
| Psychiatrist home | 12 | 0.4 (0.9) | 0 [0 - 0.5] |  | 2 | 0 (0) | 0 [0 - 0] |  |
| **Behaviour therapist (BT)** | **0** |  |  | **0** | **1** |  |  | **0** |
| BT clinic | 0 | 0 (0) | 0 [0 - 0] |  | 0 | 0 (0) | 0 [0 - 0] |  |
| BT video | 0 | 0 (0) | 0 [0 - 0] |  | 0 | 0 (0) | 0 [0 - 0] |  |
| BT phone | 0 | 0 (0) | 0 [0 - 0] |  | 0 | 0 (0) | 0 [0 - 0] |  |
| BT home | 0 | 0 (0) | 0 [0 - 0] |  | 1 | 15 (0) | 15 [15 - 15] |  |
| **Art/drama/music therapist** | **1** |  |  | **0** | **0** |  |  | **0** |
| Arts clinic | 1 | 8 (0) | 8 [8 - 8] |  | 0 | 0 (0) | 0 [0 - 0] |  |
| Arts video | 1 | 0 (0) | 0 [0 - 0] |  | 0 | 0 (0) | 0 [0 - 0] |  |
| Arts phone | 1 | 0 (0) | 0 [0 - 0] |  | 0 | 0 (0) | 0 [0 - 0] |  |
| Arts home | 1 | 0 (0) | 0 [0 - 0] |  | 0 | 0 (0) | 0 [0 - 0] |  |
| **Dietician** | **0** |  |  | **0** | **0** |  |  | **0** |
| Dietician clinic |  |  |  |  |  |  |  |  |
| Dietician video |  |  |  |  |  |  |  |  |
| Dietician phone |  |  |  |  |  |  |  |  |
| Dietician home |  |  |  |  |  |  |  |  |
| **Speech and language therapist (SLT)** | **4** |  |  | **0** | **3** |  |  | **0** |
| SLT clinic | 3 | 1.7 (1.2) | 1 [1 - 3] |  | 3 | 2.7 (2.9) | 1 [1 - 6] |  |
| SLT video | 3 | 0 (0) | 0 [0 - 0] |  | 3 | 0 (0) | 0 [0 - 0] |  |
| SLT phone | 3 | 0 (0) | 0 [0 - 0] |  | 3 | 0 (0) | 0 [0 - 0] |  |
| SLT home | 4 | 0.3 (0.5) | 0 [0 - 0.5] |  | 3 | 0 (0) | 0 [0 - 0] |  |
| **Occupational Therapist** | **4** |  |  | **0** | **1** |  |  | **0** |
| OT clinic | 2 | 1 (0) | 1 [1 - 1] |  | 1 | 1 (0) | 1 [1 - 1] |  |
| OT video | 2 | 0 (0) | 0 [0 - 0] |  | 1 | 0 (0) | 0 [0 - 0] |  |
| OT phone | 3 | 0.3 (0.6) | 0 [0 - 1] |  | 1 | 0 (0) | 0 [0 - 0] |  |
| OT home | 3 | 1.3 (2.3) | 0 [0 - 4] |  | 1 | 0 (0) | 0 [0 - 0] |  |
| **Physiotherapist (PT)** | **0** |  |  | **0** | **1** |  |  | **0** |
| PT clinic | 0 | 0 (0) | 0 [0 - 0] |  | 1 | 3 (0) | 3 [3 - 3] |  |
| PT video | 0 | 0 (0) | 0 [0 - 0] |  | 0 | 0 (0) | 0 [0 - 0] |  |
| PT phone | 0 | 0 (0) | 0 [0 - 0] |  | 0 | 0 (0) | 0 [0 - 0] |  |
| PT home | 0 | 0 (0) | 0 [0 - 0] |  | 0 | 0 (0) | 0 [0 - 0] |  |
| **Podiatry/chiropody** | **12** |  |  | **3** | **12** |  |  | **4** |
| Pod/chir clinic | 9 | 1 (0) | 1 [1 - 1] |  | 10 | 0.8 (0.4) | 1 [1 - 1] |  |
| Pod/chir video | 6 | 0 (0) | 0 [0 - 0] |  | 10 | 0 (0) | 0 [0 - 0] |  |
| Pod/chir phone | 6 | 0 (0) | 0 [0 - 0] |  | 10 | 0 (0) | 0 [0 - 0] |  |
| Pod/chir home | 9 | 0.6 (0.9) | 0 [0 - 1] |  | 10 | 0.5 (0.7) | 0 [0 - 1] |  |
| **Continence service** | **3** |  |  | **0** | **1** |  |  | **0** |
| Continence clinic | 3 | 1 (1) | 1 [0 - 2] |  | 1 | 1 (0) | 1 [1 - 1] |  |
| Continence video | 3 | 0 (0) | 0 [0 - 0] |  | 1 | 0 (0) | 0 [0 - 0] |  |
| Continence phone | 3 | 0 (0) | 0 [0 - 0] |  | 1 | 0 (0) | 0 [0 - 0] |  |
| Continence home | 3 | 0.3 (0.6) | 0 [0 - 1] |  | 1 | 0 (0) | 0 [0 - 0] |  |
| **Dentist** | **7** |  |  | **0** | **3** |  |  | **0** |
| Dentist clinic | 7 | 1.3 (0.5) | 1 [1 - 2] |  | 3 | 1 (0) | 1 [1 - 1] |  |
| Dentist video | 2 | 0 (0) | 0 [0 - 0] |  | 2 | 0 (0) | 0 [0 - 0] |  |
| Dentist phone | 0 | 0 (0) | 0 [0 - 0] |  | 0 | 0 (0) | 0 [0 - 0] |  |
| Dentist home | 2 | 0 (0) | 0 [0 - 0] |  | 2 | 0 (0) | 0 [0 - 0] |  |
| **Other health care professional** | **6** |  |  | **0** | **2** |  |  | **0** |
| Other clinic | 5 | 2 (2.2) | 1 [1 - 1] |  | 1 | 1 (0) | 1 [1 - 1] |  |
| Other video | 1 | 0 (0) | 0 [0 - 0] |  | 0 | 0 (0) | 0 [0 - 0] |  |
| Other phone | 1 | 0 (0) | 0 [0 - 0] |  | 0 | 0 (0) | 0 [0 - 0] |  |
| Other home | 2 | 0.5 (0.7) | 0.5 [0 - 1] |  | 1 | 1 (0) | 1 [1 - 1] |  |

| **Table S5 : Hospital-based health care resource use reported for individual CSRI items – all participants in both arms** | | | | | | |
| --- | --- | --- | --- | --- | --- | --- |
| **TOTAL (both arms)** | **Baseline** | | | **Follow-up** | | |
|  | **no. pts** | **Average nos. of events** | | **no. pts** | **Average nos. of events** | |
|  | **Number of participants reporting non-zero use** | **Mean visits (SD)** | **Median visits [IQR]** | **Number of participants reporting non-zero use** | **Mean visits (SD)** | **Median visits [IQR]** |
| **Hospital-based outpatient visits** |  |  |  |  |  |  |
| Psychiatric appointment | 1 | 1 (0) | 1 [1 - 1] | 0 | 0 (0) | 0 [0 - 0] |
| Epilepsy / neurology appointment | 0 | 0 (0) | 0 [0 - 0] | 2 | 1 (0) | 1 [1 - 1] |
| Specialist dental appointment | 0 | 0 (0) | 0 [0 - 0] | 0 | 0 (0) | 0 [0 - 0] |
| **General medical outpatient visits, please specify** |  |  |  |  |  |  |
| Diabetes clinic | 0 | 0 (0) | 0 [0 - 0] | 2 | 1 (0) | 1 [1 - 1] |
| ECG | 1 | 1 (0) | 1 [1 - 1] | 0 | 0 (0) | 0 [0 - 0] |
| GP | 1 | 1 (0) | 1 [1 - 1] | 0 | 0 (0) | 0 [0 - 0] |
| Kidney scan | 0 | 0 (0) | 0 [0 - 0] | 1 | 1 (0) | 1 [1 - 1] |
| Learning disabilities community team | 0 | 0 (0) | 0 [0 - 0] | 0 | 0 (0) | 0 [0 - 0] |
| Mammogram | 0 | 0 (0) | 0 [0 - 0] | 0 | 0 (0) | 0 [0 - 0] |
| MRI | 0 | 0 (0) | 0 [0 - 0] | 2 | 1 (0) | 1 [1 - 1] |
| Ophthalmology | 2 | 1 (0) | 1 [1 - 1] | 0 | 0 (0) | 0 [0 - 0] |
| Ultrasound | 1 | 1 (0) | 1 [1 - 1] | 0 | 0 (0) | 0 [0 - 0] |
| Urology | 1 | 1 (0) | 1 [1 - 1] | 0 | 0 (0) | 0 [0 - 0] |
| X-Ray | 1 | 1 (0) | 1 [1 - 1] | 0 | 0 (0) | 0 [0 - 0] |
| **Hospital admissions and visits to A&E** |  |  |  |  |  |  |
| Walk-in clinic | 0 | 0 (0) | 0 [0 - 0] | 0 | 0 (0) | 0 [0 - 0] |
| Minor Injury clinic / Urgent care centre | 0 | 0 (0) | 0 [0 - 0] | 3 | 1 (0) | 1 [1 - 1] |
| Visit to non-24-hour A&E department | 0 | 0 (0) | 0 [0 - 0] | 0 | 0 (0) | 0 [0 - 0] |
| Hospital A&E department but not admitted | 3 | 1 (0) | 1 [1 - 1] | 4 | 1 (0) | 1 [1 - 1] |
| **Visit to A&E and admitted overnight, please specify** |  |  |  |  |  |  |
| A&E / MRU (number of visits) | 1 | 2 (0) | 2 [2 - 2] | 3 | 1 (0) | 1 [1 - 1] |
| A&E / MRU (total nights per visit) |  | 2 (0) | 2 [2 - 2] |  | 2 (1.7) | 1 [1 - 4] |
| A&E / MRU (IC/HDU nights per visit) |  | 0 (0) | 0 [0 - 0] |  | 0 (0) | 0 [0 - 0] |
| General ward (number of visits) | 1 | 1 (0) | 1 [1 - 1] | 1 | 1 (0) | 1 [1 - 1] |
| General ward (total nights per visit) |  | 8 (0) | 8 [8 - 8] |  | 2 (0) | 2 [2 - 2] |
| General ward (IC/HDU nights per visit) |  | 0 (0) | 0 [0 - 0] |  | 0 (0) | 0 [0 - 0] |
| UCU (number of visits) | 0 | 0 (0) | 0 [0 - 0] | 1 | 2 (0) | 2 [2 - 2] |
| UCU (total nights per visit) |  | 0 (0) | 0 [0 - 0] |  | 3.5 (0.7) | 3.5 [3 - 4] |
| UCU (IC/HDU nights per visit) |  | 0 (0) | 0 [0 - 0] |  | 2 (2.8) | 2 [0 - 4] |
| Planned procedures | 0 | 0 (0) | 0 [0 - 0] | 0 | 0 (0) | 0 [0 - 0] |

**Table S6: Rates of use of prescribed medication including anti-dementia medication. N = number of participants reporting non-zero use, mean (median) indicates mean (median) number of prescriptions per participant, considering those reporting non-zero use.**

| **TOTAL (both arms)** | **Baseline** | | | | **Follow-up** | | | |
| --- | --- | --- | --- | --- | --- | --- | --- | --- |
|  | **N** | **Mean (SD)** | **Median [IQR]** | **N** | | **Mean (SD)** | **Median [IQR]** |  |
| **Allergy or respiratory** | 10 | 1.6 (0.9) | 1 [1 - 2] | 7 | | 1.8 (0.9) | 1.5 [1 - 3] |  |
| **Anti-dementia** | 17 | 2.1 (0.9) | 2 [2 - 2] | 14 | | 1 (0) | 1 [1 - 1] |  |
| **Antibiotic** | 2 | 1 (0) | 1 [1 - 1] | 4 | | 1.4 (0.5) | 1 [1 - 2] |  |
| **Cardiovascular** | 23 | 1.8 (0.9) | 2 [1 - 3] | 23 | | 2 (0.9) | 2 [1 - 3] |  |
| **Diabetes** | 5 | 2.1 (0.8) | 2 [2 - 3] | 5 | | 2.8 (1) | 3 [2 - 4] |  |
| **Emollient or other topical** | 14 | 2.9 (1.6) | 2 [1 - 4] | 14 | | 1.8 (1.3) | 1 [1 - 2] |  |
| **Laxative** | 8 | 1 (0) | 1 [1 - 1] | 4 | | 1.4 (0.5) | 1 [1 - 2] |  |
| **Mental health or epilepsy** | 24 | 2.9 (1.1) | 3 [2 - 4] | 21 | | 3.4 (1.6) | 3 [2 - 5] |  |
| **Painkiller** | 16 | 1.3 (0.5) | 1 [1 - 2] | 12 | | 1.2 (0.4) | 1 [1 - 1] |  |
| **Proton pump inhibitor** | 14 | 1 (0) | 1 [1 - 1] | 13 | | 1.1 (0.4) | 1 [1 - 1] |  |
| **Sleep disorder** | 2 | 1 (0) | 1 [1 - 1] | 1 | | 1 (0) | 1 [1 - 1] |  |
| **Urinary or related** | 3 | 1 (0) | 1 [1 - 1] | 4 | | 1 (0) | 1 [1 - 1] |  |
| **Vitamin or supplement** | 22 | 1.5 (0.7) | 1 [1 - 2] | 19 | | 1.4 (0.5) | 1 [1 - 2] |  |
| **Other** | 15 | 1.8 (0.9) | 1 [1 - 3] | 13 | | 1.7 (0.8) | 1.5 [1 - 2] |  |
| **OVERALL** | **33** | **9.2 (2.7)** | **9 [8 - 11]** | **32** | | **10.3 (6.3)** | **9 [6 - 11]** |  |

**Table S7: Rates of use of over-the-counter (OTC) medication.** **N = number of participants reporting non-zero use, mean (median) indicates mean (median) number of purchases per participant, considering those reporting non-zero use.**

| **TOTAL (both arms)** | **Baseline** | | | | **Follow-up** | | | |
| --- | --- | --- | --- | --- | --- | --- | --- | --- |
|  | **N** | **Mean (SD)** | **Median [IQR]** | **N** | | **Mean (SD)** | **Median [IQR]** |  |
| **Allergy or respiratory** | 1 | 1 (0) | 1 [1 - 1] | 0 | | 0 (0) | 0 [0 - 0] |  |
| **Anti-dementia** | 0 | 0 (0) | 0 [0 - 0] | 0 | | 0 (0) | 0 [0 - 0] |  |
| **Antibiotic** | 0 | 0 (0) | 0 [0 - 0] | 0 | | 0 (0) | 0 [0 - 0] |  |
| **Cardiovascular** | 0 | 0 (0) | 0 [0 - 0] | 0 | | 0 (0) | 0 [0 - 0] |  |
| **Diabetes** | 0 | 0 (0) | 0 [0 - 0] | 0 | | 0 (0) | 0 [0 - 0] |  |
| **Emollient or other topical** | 0 | 0 (0) | 0 [0 - 0] | 0 | | 0 (0) | 0 [0 - 0] |  |
| **Laxative** | 0 | 0 (0) | 0 [0 - 0] | 0 | | 0 (0) | 0 [0 - 0] |  |
| **Mental health or epilepsy** | 0 | 0 (0) | 0 [0 - 0] | 0 | | 0 (0) | 0 [0 - 0] |  |
| **Painkiller** | 1 | 1 (0) | 1 [1 - 1] | 2 | | 1 (0) | 1 [1 - 1] |  |
| **Proton pump inhibitor** | 0 | 0 (0) | 0 [0 - 0] | 0 | | 0 (0) | 0 [0 - 0] |  |
| **Sleep disorder** | 0 | 0 (0) | 0 [0 - 0] | 0 | | 0 (0) | 0 [0 - 0] |  |
| **Urinary or related** | 0 | 0 (0) | 0 [0 - 0] | 0 | | 0 (0) | 0 [0 - 0] |  |
| **Vitamin or supplement** | 3 | 3 (1.5) | 4 [1 - 4] | 2 | | 1 (0) | 1 [1 - 1] |  |
| **Other** | 0 | 0 (0) | 0 [0 - 0] | 0 | | 0 (0) | 0 [0 - 0] |  |
| **OVERALL** | **3** | **3.3 (1)** | **3.5 [3 - 4]** | **3** | | **1.5 (0.6)** | **1.5 [1 - 2]** |  |

**Table S8. Materials purchased for group sessions, including the manuals themselves. Some materials purchased for earlier sessions were re-used in later sessions (e.g. beanbags).**

| **Session** | **Item** | **Price** | **Number** | **Split across how many groups** | **Total cost (all groups)** |
| --- | --- | --- | --- | --- | --- |
| Before start | Purchase of manual | £18.00 | 4 | 4 | £72.00 |
| Introduction | Bean bags | £8.99 | 1 | 2 | £17.98 |
| 1 | Skittles | £13.99 | 2 | 2 | £55.96 |
| 3 | Large dice for My life board game | £3.99 | 2 | 2 | £15.96 |
| 4 | Play food cans | £8.49 | 2 | 2 | £33.96 |
| 4 | Food/toy boxes | £6.00 | 2 | 2 | £24.00 |
| 6 | Felt tip pens (50) | £4.99 | 1 | 2 | £9.98 |
| 8 | Card making kit | £12.99 | 2 | 2 | £51.96 |
| 9 | Toy farm animal set | £5.98 | 2 | 2 | £23.92 |
| 9 | Small vehicles | £17.99 | 1 | 2 | £35.98 |
| 11 | Pretend money | £2.75 | 2 | 2 | £11.00 |
| 12 | Large cards for number games | £5.99 | 2 | 2 | £23.96 |
| 12 | Large Dominoes | £9.99 | 2 | 2 | £39.96 |
| 12 | Large Snakes and ladders | £13.99 | 2 | 2 | £55.96 |
| 12 | Bingo | £7.49 | 2 | 2 | £29.96 |
|  | **TOTAL (all participants)** | | | | **£502.54** |
